# Preventing perinatal HIV acquisition; current gaps and future perspectives

**DOI:** 10.1097/COH.0000000000000881

**Published:** 2024-08-21

**Authors:** Beatrice Cockbain, Sarah Fidler, Hermione Lyall

**Affiliations:** a Department of Infectious Disease, Imperial College London, Imperial College NIHR BRC; b Chelsea and Westminster Hospital NHS Foundation Trust; c Department of Infectious Disease and NIHR Imperial BRC, Imperial College London, UK

**Keywords:** HIV, prevention perinatal HIV, vertical transmission

## Abstract

**Purpose of review:**

Although current treatment could eradicate vertical transmission, in 2022, 130 000 infants acquired HIV globally. HIV suppression with antiretroviral therapy (ART) transforms survival for people living with HIV (PLWH), and prevents transmission, including vertical. International guidelines recommend lifelong ART for PLWH, consequently perinatal HIV acquisition reflects implementation gaps in the HIV care cascade. We summarize these gaps, exploring potential novel approaches and therapeutic innovations towards eliminating vertical HIV transmission.

**Recent findings:**

Multifactorial challenges continue to underpin gaps in the HIV care cascade, including accessibility, availability and sustainability of HIV testing, prevention and treatment, alongside stigma, gender-based violence and poverty. Long-acting ART may be important in preventing perinatal HIV acquisition, with early data demonstrating tolerability and efficacy of injectable ART throughout pregnancy, both as HIV treatment and prevention. Carefully selected long-acting broadly neutralizing antibodies (bNAbs) matching circulating, exposing viral envelope sequences have demonstrated safety, clinical trials are ongoing to demonstrate efficacy.

**Summary:**

Emerging clinical studies should prioritize pregnant/lactating people and infants to ensure such therapies are well tolerated and efficacious. Alongside therapeutic innovation, programmatic strategies must address social and economic challenges, ensuring sustainable HIV treatment/prevention programmes and facilitating global elimination of blood-borne viruses.

## TERMINOLOGY

Vertical transmission refers to HIV transmission to infants during the perinatal period; either through pregnancy, delivery and postpartum through lactation (breast/chest feeding). This review primarily discusses and refers to pregnant and lactating individuals. We endorse the use of person-centred, nonstigmatizing, gender-inclusive language in healthcare settings according to an individual's preference [1–3]. 

**Box 1:**
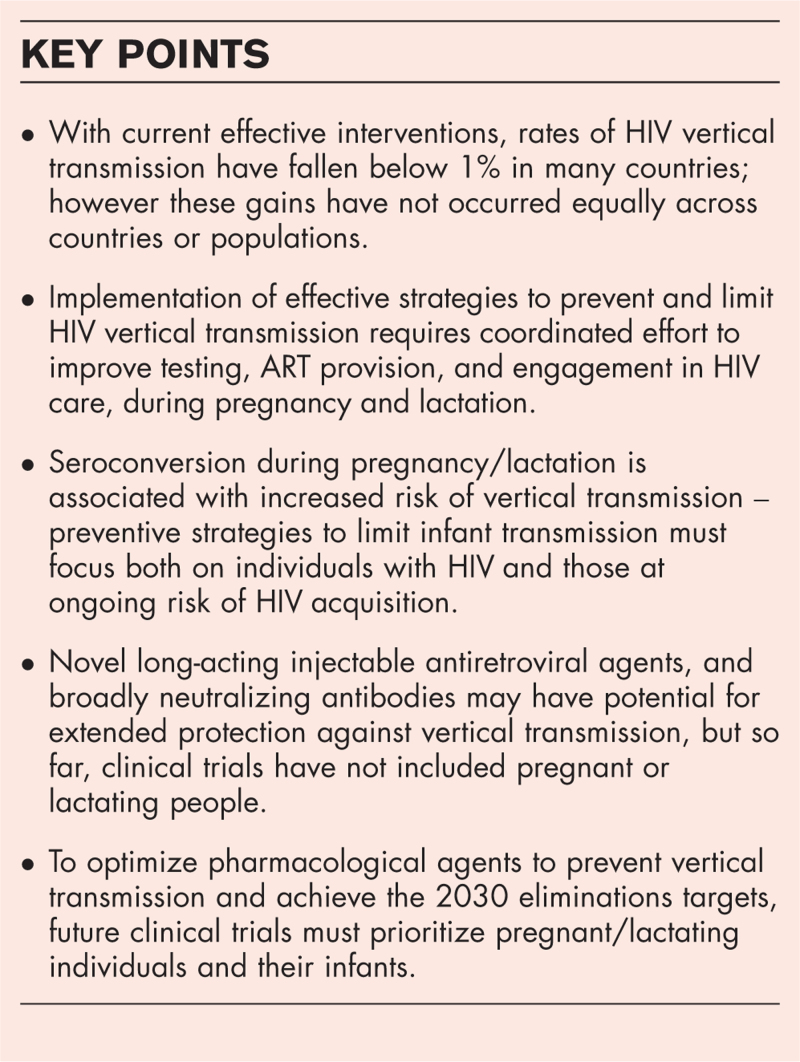
no caption available

## EPIDEMIOLOGY OF VERTICAL TRANSMISSION

Globally, 1.3 million people living with HIV (PLWH) become pregnant each year, and without interventions, the risks of in-utero, intrapartum and postnatal lactation-associated transmission are 5–10, 10–20, and 5–15%, respectively [4]. Transmission risk is greatest with seroconversion during pregnancy and lactation [5], because of high viral replication [6]. Since 2000, intervention programmes have prevented an estimated 3.4 million children (aged 0–14 years) acquiring HIV [7], although 130 000 (90 000–210 000) vertical transmissions still occurred in 2022.

The WHO's ‘triple elimination programme’ aims to eliminate vertical transmission of HIV, hepatitis B and syphilis by 2030 [8], with an HIV ‘elimination’ target of less than 5% in breast/chest-fed infants and less than 2% in formula-fed [8]. In many high-income countries, vertical transmission rates are less than 0.5% [9], whilst remaining around 12% globally (range: 9.2% in Eastern and Southern Africa; to 31.4% in North Africa and Middle East WHO regions) [10]. Since 2015, antiretroviral therapy (ART) coverage among pregnant PLWH has plateaued at 80% [10,11]; with lowest access in South East Asia (47%) and Eastern Mediterranean (23%) [10]. Th*e UNAIDS* Global Strategy 2021–2026 [12,13], and the Global Alliance to End AIDS in Children by 2030 supports priority countries to increase political commitment, action and resourcing, to eliminate paediatric HIV/AIDS [14]. Seventy-nine countries in the programme have national plans for HIV vertical transmission elimination; whereas 63 countries are integrating paediatric HIV into primary healthcare [15]. Globally, only 57% (44–78%) of children started ART in 2022, and only a third in Latin America, the Caribbean, West and Central Africa, North Africa and the Middle East [16], highlighting the importance of preventing vertical HIV transmission.

## MECHANISMS OF VERTICAL HIV TRANSMISSION

Table 1 summarizes the mechanisms of HIV transmission through the different stages of infant exposure, with viraemia the primary risk factor for transmission at all stages. In pregnant/lactating PLWH, ART reduces viraemia, and provides foetuses/infants with both preexposure prophylaxis (PrEP) and postnatal prophylaxis (PNP) via transplacental transfer [17]. Transplacental ART transfer is far higher than into milk, making milk transfer unlikely to provide sufficient ART for infant PrEP/PNP [18]. During parental seroconversion, foetuses/infants are particularly susceptible to HIV acquisition due to peak viraemia in plasma, cervico-vaginal fluids [6], and milk [5]. This observation emphasizes the necessity of regular HIV testing, for the pregnant person and their partner, with PrEP provision during pregnancy/lactation for those at ongoing HIV acquisition risk [19^▪^].

## CURRENT STRATEGIES TO PREVENT VERTICAL HIV INFECTION

### Pregnant PLWH: current prevention strategies

Pregnant PLWH on fully suppressive ART prior to pregnancy carry the lowest risk of vertical transmission. For those diagnosed with HIV during pregnancy, immediate ART initiation is recommended [20,21]. Full viral suppression before delivery, obviates the need for a prelabour Caesarean section, allowing safe vaginal delivery [22]. Sustained postnatal ART-mediated viral suppression is protective against both lactation-associated transmission, and for future pregnancies [23] and is particularly challenging for younger and newly diagnosed pregnant people [28].

ART regimens recommended for pregnant PLWH include an integrase inhibitor, usually dolutegravir, with dual nucleoside reverse transcriptase inhibitors, usually tenofovir and lamivudine or emtricitabine [24]. Global monitoring for teratogenicity is recommended via the international pregnancy registry, an essential responsibility for those prescribing ART in pregnancy [24,25]. PANNA is an ongoing pharmacokinetic study of ART use in pregnancy, when physiological changes may adversely affect drug concentrations [26]. Protease inhibitors may increase premature delivery, a balance of risk acceptable in high-income countries (HIC), but not in many low-income and middle-income countries (LMIC) [27].

### For pregnant people without HIV current prevention strategies

For those at risk of HIV acquisition, linkage to HIV prevention programmes throughout pregnancy and lactation, with encouragement to use PrEP in pregnancy [28,29] is recommended. Repeat HIV testing from 24 weeks’ gestation is recommended for people without HIV [30], with some high prevalence areas, such as South Africa, also testing at delivery [31].

#### Infant HIV testing and postnatal prophylaxis

WHO recommends nucleic acid testing (NAT) for HIV RNA/DNA in infants, as parental HIV antibody may persist until at least 18 months of age [24]. HIV detected at birth implies in-utero transmission with a high risk of rapid disease progression. HIV detected at 4–6 weeks of age will pick up most in-utero and intrapartum transmissions, with subsequent testing required for breast/chest-fed infants [24]. Lack of access to point-of-care NAT, delay in return of results, and lack of integration of HIV care with routine infant health monitoring, alongside social stigma, remain significant barriers to early infant diagnosis and initiation of ART [32].

In LMIC, WHO recommends infants at high risk of HIV transmission should receive 6 weeks of PNP (zidovudine and nevirapine), with an additional 6 weeks if breast/chest-fed [24]. In HIC, high-risk infants receive 4 weeks of triple ART (personalized according to viral resistance), with formula-feeding [33].

**Table 1:** Factors associated with risk of HIV vertical transmission

	Stage of transmission		
	*In utero*	Intrapartum	Postpartum (lactational)
Proposed mechanism of transmission	Placental exposure and subsequent infection from cell-free virus with subsequent transmission of cell-associated virus from placenta to foetus [34–36].	Exposure of infant mucosal surfaces to cell-free virus in cervical/vaginal secretions, and blood mixing during delivery [35,37].	Transmission via milk of cell-free and cell-associated virus to infant mucosal surfaces [5,35,38,39], for example, gut [40], and/or tonsils/adenoids [41]. Lactational HIV transmission may also occur through surrogate breast/chest-feeding [42]. Rarely, postnatal mode of transmission has been attributed to other postnatal practices including premastication of food [42,43].
Factors associated with transmission from birthing/lactating parent	Viraemia during pregnancy, including from seroconversion [44], is the primary risk factor for in-utero transmission [35]. The highest risks of transmission are associated with viraemia in the third trimester, with lower risks of transmission when viraemia is limited to earlier in pregnancy [45]. Inflammation/infection during pregnancy affecting integrity of birthing parent/foetal barrier and/or placenta [35]. Pregnancy co-infections and choroiditis, including with syphilis [46], CMV [47], and malaria [48]; placental inflammation from preeclampsia [49]. Altered placental integrity and vascularity [50], particularly from third trimester, increased placental blood flow in late pregnancy and placental haemorrhage [51], may also increase risk of in utero transmission [52]	Viraemia near to or at the time of delivery, including from seroconversion [44], is associated with higher risks of intrapartum transmission [45] and is a major risk factor for intrapartum transmission [35]. Factors that disrupt genital mucosal integrity, including concurrent genital infection and/or ulceration [37,53], including with HSV [53], syphilis, chlamydia, gonorrhoea [54]. Prolonged rupture of membranes [55,56], and mode of delivery (e.g. even in context of birthing parent viraemia, transmission risk significantly reduced in a prelabour prerupture of membranes Caesarean section compared with a spontaneous vaginal delivery [57])	Viraemia during lactation is the primary risk factor for postnatal HIV transmission [35]. Seroconversion during lactation is associated with the highest risk of transmission [5,44]. In paired samples, HIV viral load is usually greater in plasma than milk [58]; however, higher HIV viral loads in milk and plasma are both associated with increased vertical transmission risks [58]. Factors disrupting mucosal integrity in lactating parent, including mastitis [59,60], and cracked nipples [37]. Role for lactating parent antibodies in transmission unclear, some suggestion that persistence of lactating parent anti-HIV IgA and IgM in milk associated with reduction in transmission risk [61]; higher levels of inflammatory markers in milk associated with increased risk of transmission [62,63].
Factors associated with HIV acquisition in foetus/infant	Possible role of foetal immune response, with foetal sex differences [64,65], and HIV tropism [66], seen to affect likelihood of in utero HIV acquisition. *In utero* HIV acquisition associated with premature delivery and low birth weight [46].	Factors associated with disruption of infant mucosal barriers including use of invasive foetal monitoring, including foetal scalp electrodes [67], alongside premature delivery and low birth weight [68].	Factors associated with disruption of mucosal barrier in infant, including diarrhoeal infections and early introduction of solids associated with gut inflammation [60,68], and prematurity-associated gut dysfunction [38,68].

## GAPS IN THE HIV TESTING, PREVENTION AND TREATMENT CASCADE

Although vertical transmission is avoidable, many children still acquire HIV, understanding the gaps in prevention is key to elimination of vertical transmission.

### Antenatal HIV-testing gaps

Despite guidelines, recommending HIV testing for all, antenatal testing coverage in sub-Saharan Africa (SSA) still reaches only 63% [69]. In comparison, in the UK, 99.8% of pregnant people had an HIV test in 2022 [9].

Analysing 17 countries (83 854 people) in SSA found antenatal HIV testing positively associated with older age, urban residence, increased education, higher frequency of antenatal care visits and living in Rwanda [69]. Recent data from a South African perinatal HIV prevention programme with 31.3% antenatal HIV prevalence, reported 1.8% vertical transmissions, with high awareness of HIV status (88.3%) and ART coverage (77.9% [70]).

### Antiretroviral therapy during pregnancy and postpartum gaps

In high-burden LMIC settings, ART coverage amongst pregnant people remains sub-optimal. A Tanzanian antenatal surveillance study, where HIV prevalence is 5.9% (1.9–16.4%), identified only 66.6% [95% confidence interval (CI) 62.4–70.6%] of pregnant PLWH knew their status and were on ART [71]. In 2022, UNAIDS reported vertical elimination programmes reached 82% (64–98%) of pregnant/lactating PLWH globally [16], but there remains considerable regional variation (only18% in Asia/Pacific region) [10].

### Gaps in prevention for pregnant people without HIV

Acquiring HIV during pregnancy/lactation, is an additional gap in current practice, risking vertical transmission [10]. For pregnant/lactating people at high risk of HIV acquisition, gaps in access to PrEP remain [72]. Even where oral PrEP is freely available [19^▪^], many discontinue, with frequent seroconversions [73]. Even in those who continue PrEP use, adherence remains sub-optimal, with only 41% having therapeutic drug levels [74^▪^]. Identifying pregnant/lactating people at risk of HIV acquisition remains a significant challenge [75].

Multiple challenges limit efficacy of oral PrEP during pregnancy/lactation, including: availability, pill burden, sustained adherence, stigma, side effects and logistics, especially postpartum [76,77]. Safety monitoring of oral (tenofovir/emtricitabine) and injectable (cabotegravir) PrEP during pregnancy/lactation is ongoing, as yet no significant safety signals are reported [28,29].

A recent study of risk factors linked with HIV acquisition in pregnancy [78], included: young age (20–24 years old), informal house structure, at least two previous pregnancies, the same group, who are less likely to repeat test in pregnancy [67]. Efforts to reduce incident infection by self-testing partners of pregnant people without HIV in Zambia increased HIV testing, but not linkage to HIV care, and risked interpersonal violence [79]. Indeed, violence and harmful gender norms remain significant barriers to HIV testing, prevention and treatment [80].

### Antiretroviral therapy in pregnancy and lactation

Significant challenges to ART adherence during pregnancy/lactation remain, exacerbated by postpartum changes in HIV care provision from antenatal to routine adult services [81], and further impacted by postpartum mood disturbance and poverty [82,83]. Treatment should be linked with social and implementation strategies, which align HIV care with routine pregnancy and postpartum infant care, as promoted in the consensus Ugandan model of care (Table 2, from [33,84]). Table 2 compares models of antenatal/postnatal HIV care between two countries (Uganda and UK), both with government regulation of all healthcare facilities. Uganda, a high-prevalence, low-resource setting, is a priority country for HIV vertical elimination, compared with the UK a low-HIV prevalence, high-resource setting.

**Table 2:** Comparison of current HIV antenatal/postnatal guidelines in low-resource and high-resource settings (Uganda and UK)

	Uganda – mainly breast/chest-feeding Elimination: <5% transmission rate Latest guideline 2022		UK – mainly formula-feeding Elimination: <2% transmission rate Latest guideline due in 2024 (latest published guideline 2018 with 2020 update)	
Testing	HIV/HBV/syphilis test at first antenatal visit, third trimester, and delivery. Use same-day result tests Routine offer of PrEP to all seronegative women		HIV/HBV/Syphilis test at first antenatal visit, takes 7–10 days to get result. Only test again if new infection risk ascertained. No routine offer of PrEP to seronegative women	
Transmission risk	High risk	Low risk	High risk	Low risk
Pregnancy	If not on ART start same day as test, use INSTI-based ART, aim for VL <200 ASAP, 3 monthly VL monitoring. Instigate MDT support	If on ART, maintain VL <200, 3 monthly VL monitoring. Instigate MDT support	If not on ART, baseline resistance and start ASAP, use INSTI-based ART, aim for VL <50 ASAP, monthly VL monitoring. Instigate MDT support. If poor adherence, check resistance and TDM	Maintain VL < 50, if on ART at conception do not change, unless ART with poor PK in pregnancy (e.g. cobicistat/etravirine)
Delivery	SVD as per safe obstetric practices Continue daily ART	SVD as per safe obstetric practices Continue daily ART	VL> 1000 – elective caesarean section and IV ZDV. VL 50–1000 – consider caesarean section and IV ZDV. Continue daily ART	SVD as per safe obstetric practices Continue daily ART
Infant feeding	Optimal BF practices, avoid solids before 6 months, MDT and adherence support		VL > 50 at delivery/adherence issues Advise FF – support with FF	Sustained VL < 50 before and after delivery – support FF or BF, as per parental choice.
Infant PNP	NVP 12 weeks	NVP 6 weeks	ZDV/NVP/3TC 4 weeks (different agents, if known parental viral resistance)	ZDV 2 weeks
Infant diagnosis	HIV DNA – 4–6 weeks, 9 months, then 6 weeks after stopping BF, then HIV Ab at 18 months. Link to routine visits for immunizations. If possible, use same day tests. If new maternal HIV diagnosis during BF – start infant triple ART, until confirm infection or not.	HIV DNA - 4–6 weeks, 9 months, then 6 weeks after stopping BF, then HIV Ab at 18 months. Link to routine visits for immunisations. If new maternal HIV diagnosis during BF – start infant triple ART, until confirm infection or not	HIV DNA & RNA - 2–3, 6, and 12 weeks, then HIV Ab at 22 months	FF – HIV RNA – 6,12, weeks, then HIV Ab at 22 months. BF - HIV RNA – monthly during BF, then 4 and 8 weeks after stopping BF, then HIV Ab at 22 months
Integration	Link to routine visits for immunisations.			

Data from [33,84]. 3TC, lamivudine; Ab, antibody; ART, antiretroviral therapy; ASAP, as soon as possible; BF, breast/chest feeding; CPT, co-trimoxazole preventive therapy; FF, formula feeding; HBV, hepatitis B virus; INSTI, integrase inhibitor; ITNs, insecticide-treated nets; i.v., intravenous; MDT, multidisciplinary team; NVP, nevirapine; PITC, Provider-initiated HIV testing and counselling; PK, pharmacokinetics; PNP, postnatal prophylaxis; PrEP, HIV preexposure prophylaxis; SVD, spontaneous vaginal delivery; TDM, therapeutic drug monitoring; VL, HIV viral load, measured in copies per millilitre; ZDV, zidovudine (AZT).

### Gaps in infant postnatal prophylaxis

Transplacental ART protects infants in the first hours/days after delivery, so first-line adult ART should include agents that efficiently cross the placenta, with a half-life of at least 48 h in the neonate (e.g. dolutegravir or tenofovir). This is particularly important for premature or sick infants who cannot take enteral medication immediately after birth.

Infant PNP, still depends on older agents, including nevirapine, a nonnucleoside reverse transcriptase inhibitor (NNRTI), where prevalence of HIV-1 NNRTI-resistance in SSA is 10–50% [85,86]. To date, the median time to regulatory approval for ART for neonates is 8 years after adult approval (range 2–23 years) [87]. Neonatal pharmacokinetic studies of dolutegravir have only just been reported [88], and it will take some time before full dosing data is available. Access to newer agents, including injectables, should be prioritized for infants [87].

### Programmatic funding gaps

International funding for HIV programmes has been static since 2013, with a funding deficit of $8.5 billion to achieve ART coverage targets by 2025 [7]. Lack of universal ART coverage is of particular relevance for people of reproductive age who represent two-third of new HIV diagnoses in SSA [10]. Access to ART is the primary driver of vertical transmission in western/central Africa [10], whereas for eastern/southern Africa, engagement in care remains the primary challenge [10].

Beyond funding, additional challenges to ending HIV include conflict and climate change, displacing 114 million people forcibly by September 2023 [89], with 20% in the eastern, Horn and Great Lakes regions of Africa [89]. Although some countries report equivalent HIV care provision for refugees [90], this is not universal, and may be limited by other legislation, particularly in relation to gender/sexual minorities [91]. Further, reductions in HIV care through the COVID-19 pandemic have still not recovered [10].

### Novel therapies and strategies to cover the gaps

Implementation studies aimed at improving HIV prevention strategies for both men and women, HIV testing, linkage to care maintained through the postpartum period and ART adherence for this vulnerable population are required.

In two randomized trials, self-testing for partners and pregnant people was effective in reducing vertical transmission [79]. Closing the ‘postpartum’ gap when PLWH are referred from antenatal clinics back to routine ART services, requires targeted or combined infant–parental clinics to ensure maintenance on ART [10,92]. Support with adherence during pregnancy/postpartum can been enhanced through peer support programmes such as ‘mother-to-mother’ [93]. Amalgamation of adolescent Sexual and Reproductive Health and HIV services can ensure access to HIV testing/treatment/prevention services, without barriers, including the need for parental consent [94,95].

Long-acting antiviral agents for pregnancy/postpartum, for both parent and infant, most likely have a significant future role to improve adherence. Injectable ART agents; cabotegravir with rilpivirine, lenacapavir and islatravir, are under investigation as long-acting treatments as well as prophylaxis, although globally access maybe limited by cost [96]. Passive immunization with long-acting HIV-specific broadly neutralizing antibodies (bNAbs), have been demonstrated to be well tolerated in adults and infants, and are currently under investigation for prevention of vertical transmission [97^▪▪^].

#### Novel therapies for pregnant people without HIV at risk of HIV acquisition

The safety of oral PrEP in pregnancy has been demonstrated in numerous studies [30,98], but oral PrEP has not reduced HIV incidence in southern Africa to the same extent as in other countries, primarily because of low adherence, insufficient scale-up of PrEP services, and stigma amongst at-risk groups (clinical trials summarized in Table 3). However, access to long-acting injectable ART for prevention in this context could deliver a significant impact on these challenges. The HPTN084 study demonstrated the efficacy of injectable cabotegravir to prevent HIV acquisition amongst cis-women living in sub-Saharan Africa (SSA), and included 39 pregnant people, reporting no congenital abnormalities [99]. However, there is still only limited data on safety of long-acting cabotegravir in pregnant/lactating individuals without HIV. To date, unfortunately, there are no registered trials of cabotegravir in pregnancy/lactation, or as infant PNP.

In addition to injectable agents, the monthly applied dapivirine vaginal ring has been tested for acceptability, safety and efficacy when compared with oral PrEP amongst 247 young women without HIV in a randomized controlled trial in SSA [100]. The vaginal ring was well tolerated, acceptable and safe, with a comparable profile to oral PrEP, although overall adherence to either modality was low (57%) and no pregnancy outcomes were reported [100]. Subsequent trials of the dapivirine ring as PrEP during pregnancy (MTN-042/DELIVER [101]) and lactation (MTN-043-B-PROTECTED [102]) reported good safety profiles and, when used during lactation, low milk transfer into infants [102]. Indeed, a 2024 meta-analysis of oral PrEP and the dapivirine ring in pregnancy/lactation found no evidence for adverse perinatal outcomes associated with these methods, although no data was available for injectable PrEP [103]. Several studies that aim to test safety efficacy and pharmacokinetics of long-acting injectables, the dapivirine vaginal ring and novel small molecules have been identified in a recent review [104], and should be prioritized within clinical research.

#### Long-acting antiretroviral agents for HIV prevention

Rilpivirine/cabotegravir – while injectable long-acting ART (LA-ART) has regulatory approval for PLWH in many settings, there is limited data on pharmacokinetics and effectiveness for prevention of vertical transmission among pregnant PWLH [105]. Unfortunately, the recently reported CARE trial, which assessed implementation of LA-ART in SSA excluded pregnant PLWH [106]. A report of 25 pregnancies, with 10 live births (one case of congenital ptosis), amongst PLWH enrolled in LA-ART trials or receiving LA-ART for compassionate grounds, were included in a recent review [107]. This study demonstrated pharmacokinetics similar to nonpregnant people.

Islatravir [108], is a first-in-class nucleoside reverse transcriptase translocation inhibitor with a long half-life supporting its use as a long-acting option for PrEP. A once-monthly oral dose of islatravir maintains effective concentrations of its active metabolite. An investigational implantable formulation may provide efficacious concentrations for up to a year, with comparable distribution into vaginal and rectal tissues making it a promising PrEP option. In rhesus macaques, weekly oral islatravir provided complete protection, following intrarectal challenge with simian-human immunodeficiency virus (SHIV) [109]. Human PrEP studies remain at an early stage and exclude pregnant/lactating people. The Impower-022 islatravir PrEP study is currently paused because of concerns around falling lymphocyte count [110], an issue of particular concern if used in pregnancy, given the relative immunosuppression of this period.

Lenacapavir, a potent capsid inhibitor, with demonstrated safety and efficacy following twice-yearly subcutaneous dosing in PLWH, is approved for treatment of multidrug-resistant HIV-1 [111]. In nonhuman primates, lenacapavir prevents SHIV infection following intrarectal challenge [112,113]. No studies have proven its efficacy in preventing vertical transmission. There are currently two listed ongoing clinical trials of lenacapavir PrEP for cis-women [114,115] but excluding pregnant people.

#### Passive immunization for infants at risk of HIV acquisition with HIV-broadly neutralizing antibodies

Infant prophylaxis with long-acting, injectable bNAbs could provide protection from vertical HIV transmission throughout lactation [116]. BNAbs have demonstrated efficacy as postnatal prophylaxis (PNP) in nonhuman primates, including postexposure efficacy against intrapartum transmission if given within 24–30 h after birth [117]. The HPTN-AMP trial amongst HIV− women in SSA tested several monthly intravenous doses of bNAb VRC01 [118]. Whilst VRC01 was safe, well tolerated, acceptable and feasible, overall, it did not significantly prevent sexual HIV transmission compared with standard of care. However, VRC01 did protect against transmission of antibody-sensitive HIV-1 variants. The key lesson from the AMP study is that selection of bNAbs for prevention must match the exposing viral variants. For exposed infants, this is much easier to establish where parental HIV-1 envelope sequencing is potentially available. Implementation of this complex technology in resource-limited settings maybe challenging. Exposure to a single bNAb carries the risk of rapid development of viral resistance, where models predict the driving factor of antibody resistance is antibody dose and viral load, leading to lack of efficacy [119].

Long-acting Fc-modified bNAb variants are now in trial, and intravenous infusions confer therapeutic levels of antibody for 6–9 months, making this approach well suited to prevention of vertical transmission during lactation [97^▪▪^]. Other and combined bNAbs (e.g. VRC01LS and VRC07-523LS) have demonstrated safety, tolerability and potentially protective levels for 3 months when given subcutaneously to HIV-exposed uninfected infants soon after birth and during infancy [97^▪▪^]. Wherever possible, bNAb regimens for exposed infants without HIV could be linked to the routine infant vaccination schedules to simplify care pathways. Future studies must also confirm that HIV bNAbs do not interfere with normal infant vaccine responses. A cost-effective modelling paper in South Africa, Zimbabwe and Cote D’Ivoire identified that adding long-acting bNAbs to current standard-of-care infant prophylaxis would be cost effective [120]. In all three settings, bNAb strategies would remain cost-effective at costs up to $200 per dose, if efficacy is at least 30%. Lastly, if demonstrated to be efficacious as adult HIV treatment/prevention, injectable bNAbs could be beneficial to those struggling to use oral medications during pregnancy/lactation.

**Table 3:** Summary of novel approaches to limit vertical HIV transmission under investigation

New therapies	Trial data to support efficacy	Mechanism of action
HIV-specific broadly neutralizing antibody to prevent infants without HIV who have been exposed to HIV-acquiring HIV (‘HIV-exposed uninfected’ infants)	VRC01 phase I human (NCT02256631). A single subcutaneous dose of VRC01 was administered in combination with standard antiretroviral prophylaxis to formula-fed and as multiple doses to breastfed HIV-exposed infants and shown to be safe and well tolerated [121] IMPAACT 2008 (NCT03208231) 40 mg/kg × 4 doses s.c. VRC01 *n* = 30 reported grade 1 mild safety issues only (ongoing) [122,123] IMPAACT P1115 (NCT02140255) VRC07–523LS 40 mg/kg × 1 dose s.c. *n* = 179 (ongoing) [122,124] IMPAACT P1112 (NCT02256631) open-label dose-escalation phase I trial amongst HIV-exposed infants without HIV of single sub-cutaneous administration of VRC01-LS and VRC07–523LS *n* = 83 infants second dose for those still breast feeding at 6 months [122,125] IMPAACT 2037 (DAIDS registration number 38962) PGT121.414LS +/- VCR07–523LS (ongoing) [126] PedMAb 1 CAP254V2LS +/- VRC07–523LS South Africa [127]	Direct antiviral, ADCC and antibody-mediated immune modulation [128]
Injectable antiretroviral therapy	Cabotegravir Limited safety data is available for pregnant/lactating PLWH. To date, 10 live infants have been born to PLWH on cabotegravir/rilpivirine ART with one reported congenital abnormality [107]. Islatravir Limited data for ART in cis-women and no data in pregnancy or for infant PNP. To date, the one study in cis-women is currently paused (NCT04644029 [110]).	Long-acting injectable ART for pregnant PLWH

ART, antiretroviral therapy; PLWH, people living with HIV.

## DISCUSSION

There is much optimism around long-acting agents to prevent vertical HIV transmission, especially for those who struggle with adherence to daily oral medication. However, the cost of implementing LA-ART maybe prohibitive in many settings [129]. Furthermore, the high global prevalence of HIV resistance to NNRTIs [130], and increasing integrase inhibitor resistance, up to 4–19% in some high burden settings [130], challenges widespread future efficacy of rilpivirine/cabotegravir LA-ART. New ART such as lenacapavir [131], and islatravir [132], are potentially more practical agents for HIV prevention and treatment, but with no data yet on safety and efficacy for prevention of vertical transmission.

Long-acting bNAbs have been widely tested in early-phase clinical trials (although largely amongst cis-men living with HIV), demonstrating safety and efficacy at maintaining viral suppression off ART [133], however only among those with virus sensitive to the bNAb HIV envelope-binding site [134]. Predictive assays for bNAb sensitivity, include envelope sequencing and viral phenotype, are in development but remain sub-optimal, time consuming and expensive [135]. The immune modulatory mechanism of action of bNAbs is highly pertinent to prevention of vertical transmission, as not only do bNAbs act as direct antiviral agents but also induce HIV-specific immune responses, the so-called ‘vaccinal’ effect [136], which may confer extended viral suppression even after bNAb levels are reducing. The need for virus-specific bNAbs to effectively treat and prevent vertical transmission for an individual person means that widespread use will take some time to scale-up before it can become cost effective [137]. New studies are underway to test the strategy, and rationale for this approach, which has the potential for impact, especially in the setting of increased ART resistance [138^▪▪^]. Long-acting immune-modulatory effects of carefully selected bNAbs could potentially confer lasting protective immunity against transmission.

Although long-acting agents do offer hope for the future, improved implementation of current strategies is essential to eliminate perinatal HIV acquisition. Despite complexities within the cascade of care for pregnant/lactating PLWH, a number of LMICs have been conferred ‘Triple Elimination Status’ for HIV, hepatitis B and syphilis [139]. Demonstrating that with current interventions, successful implementation of prevention transmission is feasible, even with constrained resources. Recent USA data demonstrates the necessity within a country, to improve equity of access across all ethnic/racial groups, to ensure that elimination targets are equally and equitably achieved [140]. Pregnant people and infants are recognized by the WHO as one of the ‘Key HIV Susceptible Populations’ [141], maintaining annual epidemiological surveillance, and prioritizing their immediate access to the fully supported cascade of HIV care could even now enable elimination of perinatal HIV. Table 4 summarizes key review article findings.

**Table 4:** Key review article findings

Challenge	Recommendation
Testing gaps	Repeat testing during pregnancy Partner testing Test infants at birth, and during and after breast/chest feeding
ART coverage gaps for pregnant/lactating PLWH, and for those without HIV at risk of seroconversion during pregnancy/lactation	Improved access to ART and PrEP throughout pregnancy/lactation Improved adherence support for both ART and PrEP Enhanced access to nonoral formulations of ART/PrEP during pregnancy and lactation Inclusion of pregnant/lactating people in ART/PrEP trials to increase safety/efficacy/pharmacokinetic data
Weak points in the HIV care cascade for ART coverage	Improved transition between antenatal and routine HIV care Improved transition for PrEP access between antenatal and postnatal care
Adherence to oral ART during pregnancy/lactation for PLWH	Enhance access to injectable LA-ART for pregnant/lactating PLWH Consider novel therapies, including bNAbs at delivery for PLWH struggling with poverty, violence and issues around ART adherence, to enhance virological control throughout lactation
Access and adherence for infant PNP, including challenges with use of oral medications	Consider LA-injectable PrEP and/or novel therapies, including bNAbs, at delivery and throughout lactation for infants without HIV born to PLWH without virological suppression or at risk of loss of virological suppression.

ART, antiretroviral therapy; bNAbs, broadly neutralizing antibodies; PLWH, people living with HIV; PrEP, preexposure prophylaxis.

## CONCLUSION

Although new long-acting interventions may bring future benefit, implementation of current HIV testing with linkage to prevention or treatment cascades remains the key for elimination of vertical transmission. Pregnant/lactating PLWH and their infants should be first in line for future clinical trials, with prioritized access (if found effective) to LA-ART and passive immunization with HIV-specific bNAbs.

## Acknowledgements

*None.*

### Financial support and sponsorship

*None.*

### Conflicts of interest

*B.C. is in receipt of a National Institute of Health Research (NIHR) Academic Clinical Fellowship. S.F. is supported by the Imperial College NIHR Biomedical Research Centre and has received grant money to her institution from Viiv, Gilead, and AbbVie.*

## REFERENCES AND RECOMMENDED READING

Papers of particular interest, published within the annual period of review, have been highlighted as:

▪ of special interest
▪▪ of outstanding interest
